# Laser Rescanning for Enhancing Mechanical Properties of Laser-Directed Energy-Deposited High-Manganese Steels

**DOI:** 10.3390/mi15020176

**Published:** 2024-01-24

**Authors:** Young Keun Park, Hyun Ji Nam, Yong Ho Park, Wookjin Lee

**Affiliations:** School of Materials Science and Engineering, Pusan National University, Busan 46241, Republic of Koreaskaguswl2@pusan.ac.kr (H.J.N.)

**Keywords:** additive manufacturing, laser-directed energy deposition, high-manganese steel, laser rescanning

## Abstract

This study investigates the effects of laser deposition and laser rescanning (LR) on the microstructure and mechanical properties of high-manganese steel (HMnS) deposited by laser-directed energy deposition (L-DED) comprising 24 wt.% Mn. Four types of laser deposition and LR strategies were investigated: unidirectional L-DED scanning without laser rescanning, L-DED scanning with 90° alterations in the laser scanning path on each layer without laser rescanning, unidirectional L-DED with laser rescanning in the same direction, and L-DED with laser rescanning with 90° alterations in the laser scanning path. The L-DED-processed HMnS had only a few small pores and exhibited a microstructure without any serious defects such as cracks. Additionally, a strong fibrous texture along the <101>/building direction of the fully austenite phase was found. The mechanical properties (microhardness and tensile strength) of HMnS were improved by the LR with a grain refinement effect and fine solidification cell size due to the significantly faster solidification rate in LR than that in L-DED.

## 1. Introduction

In additive manufacturing (AM), raw materials are supplied through the computer-aided design of the desired product shape and finally deposited in the form of the product using a heat source [1,2,3]. AM is advantageous in manufacturing complex products that are difficult to manufacture using conventional casting and cutting processes. Among the AM techniques, laser-directed energy deposition (L-DED) is a method of depositing three-dimensional complex shapes by irradiating metal powders with a laser. A melt pool is formed locally where the laser is irradiated, and the molten metal powder directly supplied to the melt pool is rapidly solidified and deposited. The deposition rate in the L-DED process is generally significantly faster than that in the laser–powder bed fusion process. Additionally, it is advantageous because the nozzle through which the laser and metal powder are supplied can be moved freely, and it is not limited by the size or shape of the deposited product. Therefore, the L-DED process can be used for various purposes, such as surface strengthening, repairing metal parts, and manufacturing small or large metal parts, including micromachines and microdevices [4,5,6,7,8]. 

High-manganese steel (HMnS) is an alloy comprising 3–27 wt.% of manganese and has excellent mechanical properties, such as high strength, wear resistance, high formability, and toughness. Low-carbon, high-manganese steel containing 15–30 wt.% of manganese has excellent formability and excellent wear resistance due to high work-hardenability [9,10]. However, several difficulties related to the application of HMnS in various industries still exist because of disadvantages, such as segregation due to high manganese content, limited processability, and low formability at high temperatures. Such segregation can be reduced by the high cooling rate of L-DED, as demonstrated by Collins et al. [11]. Based on advantages such as a rapid cooling rate generated in the L-DED process, these problems of HMnS can be solved.

Additionally, Li et al. [12] developed a repair process for wear-resistant cast iron parts using a specially designed NiCu and Fe-36Ni (wt.%) alloy system using a metal arc welding process. The study indicated that worn components made of wear-resistant iron can be effectively repaired if wear-resistant materials such as HMnS can be deposited by welding in the L-DED process on the worn surface. As shown by Zhang et al. [13], HMnS is a wear-resistant alloy with a single austenite phase in structure and excellent toughness. The remarkable work-hardening ability of HMnS enables its surface to form a strong hardening layer when deformed by impact energy, which makes HMnS an attractive material for the surface hardening of impact-resistant parts or repairing worn components. The excellent bonding properties between cast iron parts and L-DED-processed HMnS can also be used to fabricate complex microdevices and micromachines. For instance, the functional features of microdevices can be realized by the L-DED of HMnS followed by the fabrication of the main part of the device by micro-casting cast iron.

In L-DED, the thermal gradient and product quality are significantly affected by the laser scanning strategy. Liu et al. [14] reported that when Inconel 718 alloy was deposited by a laser solid forming process, the grain size uniformity of unidirectional raster scanning was reduced compared to that of transverse raster scanning, but they had rather similar ultimate tensile strengths. Rombouts et al. [15] reported that the mechanical properties of Inconel 625 alloy produced by the L-DED process resulted in lower yield strength of vertically deposited specimens than that of horizontally deposited specimens. It was reported that as the L-DED process progressed, the vertically deposited specimens were deposited higher than the horizontally deposited specimens, and the solidification rate was lowered due to higher heat accumulation inside.

Laser rescanning (LR) is a process to obtain rapidly solidified microstructures. A solidified layer is remelted and solidified by LR before the deposition of the next layer. The LR process is mainly used for clad materials or coatings that improve mechanical properties, because a microstructure can be achieved through rapid solidification [16,17,18]. LR research in AM has been reported extensively in the selective laser melting process. Kenel et al. [19] demonstrated that the selective laser melting of oxide-dispersion-strengthened Ti–Al can significantly reduce the frequency of cracks generated during the LR process. Considering the mechanical properties of selective lase- melted Al–Mg–Zr alloys, Griffiths et al. [20] reported a decrease in the grain size with an increasing number of LR cycles. Regarding the L-DED process, Kim et al. [21] reported that the strength and ductility of AISI 316L stainless steel deposited by L-DED were increased by LR. 

In a pervious study by the authors, HMnS with a manganese content of 12 wt.% was successfully fabricated by the L-DED process [22]. The results showed that L-DED can produce HMnS with a near defect-free microstructure, without any significant macroscopic segregation of Mn due to rapid solidification. However, to the best of our knowledge, the effect of LR on HMnS deposited by the L-DED process has not been reported. Therefore, this study aims at investigating the effect of LR on the microstructure and mechanical properties of HMnS deposited by the L-DED process containing 24 wt.% manganese.

## 2. Materials and Methods

**Figure 1:**
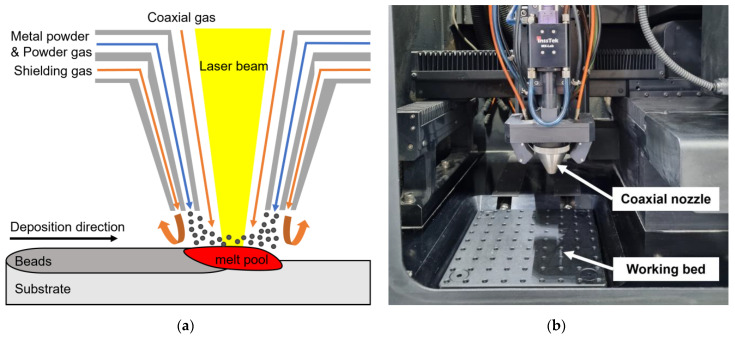
(**a**) Schematic of the L-DED process and (**b**) experimental setup.

In this study, gas-atomized spherical high-manganese Fe–24Mn–4Cr–0.4C (wt.%) steel powders with a diameter of 80–120 μm were used in the L-DED process to obtain deposited HMnS. Figure 1a shows a schematic of the L-DED process. Figure 1b shows a picture of the L-DED equipment used in this study. The L-DED process was performed using MX–Lab (InssTek Inc., Daejeon, Korea) equipped with a 500 W fiber laser. HMnS powder and carrier gas were simultaneously injected into the molten pool generated by the laser during the process.

The L-DED process and LR parameters selected in this study are listed in Table 1. The laser power, scanning speed, and powder feed rate were carefully adjusted to minimize porosity and crack formation. For this, preliminary experiments with different laser powers with a laser power interval of 40 W were performed. It was found that when deposition was performed with a laser power lower than 150 W, several lack-of-fusion pores and cracks were formed due to insufficient melting. By contrast, when deposition was performed with a laser power higher than 190 W, near fully dense samples were obtained. Therefore, considering the fact that the excessive laser heat input can significantly vaporize Mn during the process, the laser power was set to 190 W in this study. These process parameters were produced an almost completely dense HMnS alloy layer with few defects. Additionally, LR was also conducted with the same laser power and scanning speed as those in the L-DED process.

**Figure 2:**
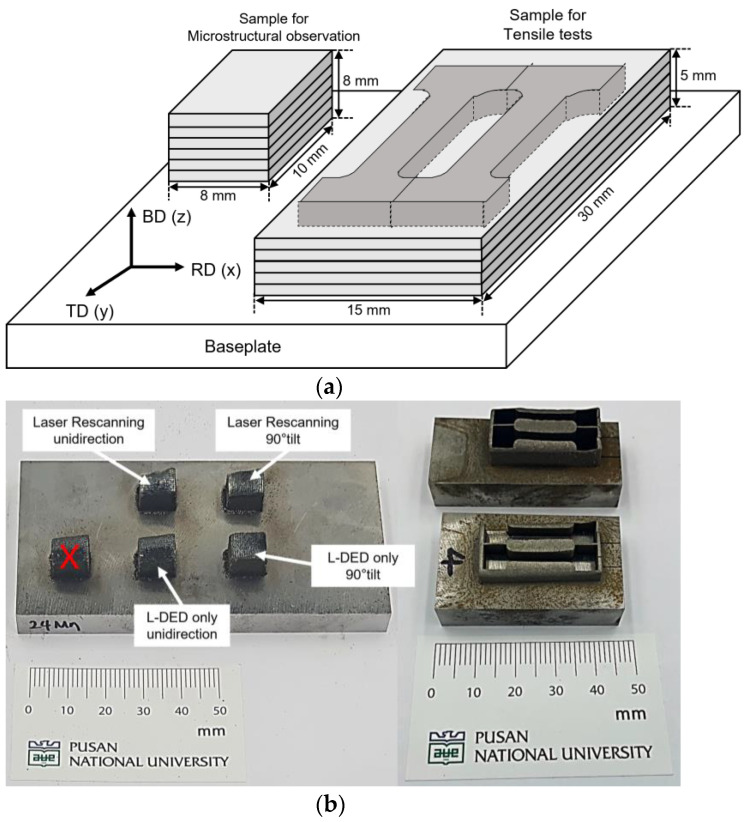
(**a**) Schematic of the sample geometry and (**b**) appearances of the deposited HMnS samples.

Figure 2a shows a schematic of the deposited samples in this study. Figure 2b shows the appearance of the deposited HMnS. Ar gas (99.999%) was used to prevent oxidation of the melt pool generated during the L-DED process and to supply HMnS powder. In the L-DED process, a medium carbon steel plate with a size of 50 × 100 × 10 mm^3^ was used as a substrate, and HMnS with two sizes of 8 × 10 × 8 and 15 × 30 × 5 mm^3^ were deposited for microstructural observation and tensile tests, respectively. The sample sizes in this study were carefully selected to avoid heat accumulation and its influence on the local microstructure during the L-DED process. Through a preliminary study, it was found that the microstructure and hardness of the bottom and top layers were nearly identical when the sample size was larger than 8 × 10 × 8 mm^3^. Samples for microstructure analysis were deposited for each condition, and the same samples were used for Vickers hardness testing. Samples for the tensile test were deposited in sizes of 15 mm × 30 mm × 5 mm, and four tensile test specimens for each condition were produced and subjected to the tests. In Figure 2b, a feature marked as ‘X’ on the photograph is the sample produced with a laser power of 150 W. This feature was not used for testing due to internal defects.

**Figure 3:**
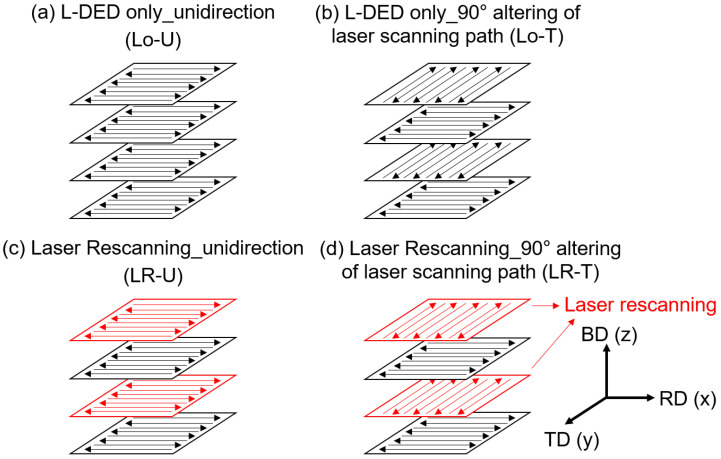
Schematics of L-DED and rescanning strategies for deposited samples: (**a**) Lo–U, (**b**) Lo–T, (**c**) LR–U, and (**d**) LR–T.

Figure 3 shows four types of laser deposition and LR strategies used in this study as follows:

1. Unidirectional L-DED scanning without laser rescanning (Lo–U, Figure 3a).
2. L-DED scanning with 90° alteration in laser scanning path on each layer without laser rescanning (Lo–T, Figure 3b).
3. Unidirectional L-DED with laser rescanning in same direction (LR–U, Figure 3c).
4. L-DED with laser rescanning with 90° alteration in laser scanning path (LR–T, Figure 3d).

Among the different possible altering angles that can be used to manipulate the microstructures and properties, the 90° altering strategy had been most frequently used in the literature, because it can effectively reduce in-plane anisotropy without any further complexity arising from controlling the laser scanning trajectory [23,24]. Thus, 90° altering was used for altering the laser scanning direction in the laser scanning strategies used in this study. 

For microstructure analysis, the deposited HMnS samples were cut into planes parallel to the deposition direction, and the surface was polished with a 0.05 μm alumina slurry. Chemical etching was performed with an aqueous solution of nital (5% nitric acid + ethyl alcohol). The microstructure was observed using an optical microscope (OM, Axio Lab 5, ZEISS, Gina, Germany) and a scanning electron microscope (JSM7200F, JEOL, Akishima City, Japan) equipped with electron backscatter diffraction (EBSD, Oxford NordlysNano, Abbington upon Thames, UK). The Vickers microhardness (HM–100, Mitutoyo Corp., Kawasaki City, Japan) was measured ten times for each specimen. The hardness values excluding the maximum and minimum values were averaged. Tensile specimens were cut in the form of dog bones according to the KS B 0801 standard [22]. The tensile test was performed at a speed of 0.1 mm/min using a uniaxial tensile testing machine (UNITECH–T, R&B Inc., Daejeon, Korea) on more than two specimens from each sample.

## 3. Results and Discussion

### 3.1. Microstructures

**Figure 4:**
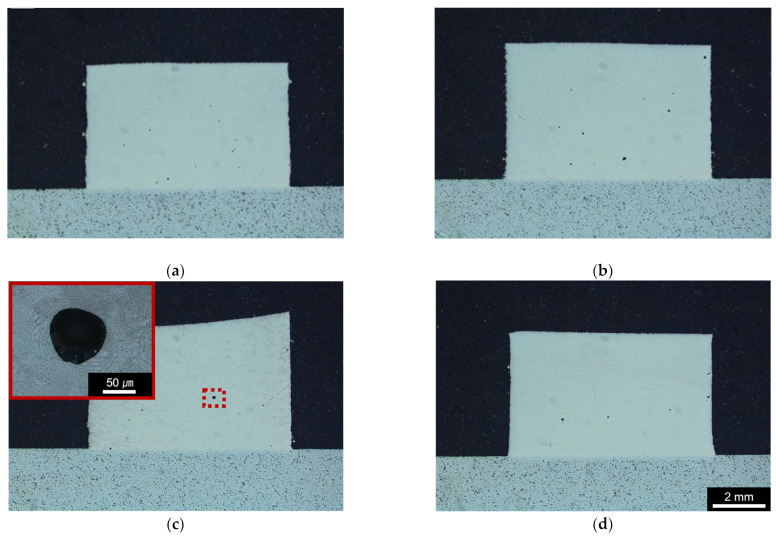
OM images of HMnS samples. (**a**) Lo–U, (**b**) Lo–T, (**c**) LR–U, and (**d**) LR–T.

Figure 4 shows the macroscopic OM images of HMnS deposited by the L-DED process by applying four types of laser deposition and LR strategies. It shows that HMnS samples deposited by the L-DED comprised near fully dense microstructure. No lack-of-fusion pores were observed for the samples produced by all four laser scanning strategies. Additionally, it was found that there were negligible defects such as cracks in the vicinity of the interface between the substrate and L-DED-deposited materials. The samples had a few small spherical pores, with a maximum size of approximately 50 μm. The red dotted box in Figure 4c shows a magnified image of the spherical pore found in the deposited HMnS sample. These spherical pores may be attributed to the gas atomization process of the high-Mn steel powder used for L-DED deposition. The porosities of the samples observed in the OM images shown in Figure 4 were quantified using ImageJ software. The results indicate very low porosities of approximately 0.60, 0.41, 0.39, and 0.68 % for the Lo-U, Lo-T, LR-U, and LR-T samples, respectively. There appears to be no significant difference in porosity along the horizontal and vertical directions of each sample.

In Figure 4c, the macroscopic cross-section image of the LR–U sample indicates a slightly sloped top surface produced during the L-DED process. This was probably due to the more pronounced heat accumulation near one edge during the process of the LR-U sample, caused by the same laser DED and rescanning tool paths used in the process. For this reason, the deposition height of the LR-U sample was higher near one edge in comparison to those at the other edges, as shown in Figure 2b. The other samples exhibited approximately flat top surfaces due to the distributed heat input.

**Figure 5:**
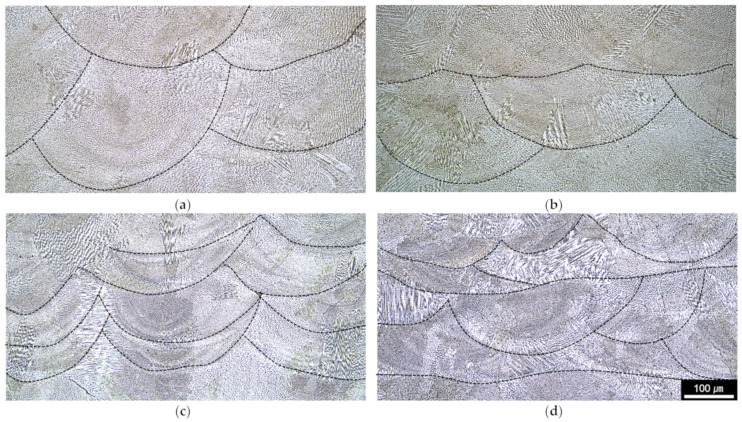
OM microstructures of samples deposited with HMnS: (**a**) Lo–U, (**b**) Lo–T, (**c**) LR–U, and (**d**) LR–T.

Figure 5 shows the OM microstructural images and X-ray pole figures of the HMnS samples. The microstructures were obtained from the cross-section at the middle of each sample on the BD-RD planes. In the OM microstructures, the laser melt pool boundaries and solidification cell structures are clearly observed. It can be seen that the laser melt pool heights of the samples with the laser rescanning (that is, LR–U and LR–T shown in Figure 5c,d) are pronouncedly smaller than those for the samples produced by L-DED only (that is, Lo–U and Lo–T shown in Figure 5a,b). The averaged melt pool heights of the samples with and without the LR process were approximately 184 and 75 μm, respectively. The laser melt pool formed by LR is smaller than that by L-DED. This is probably due to the relatively fast heat release into the surrounding solidified dense material in comparison to that to the atmosphere at one side of the melt pool in the case of normal L-DED [20,21].

**Figure 6:**
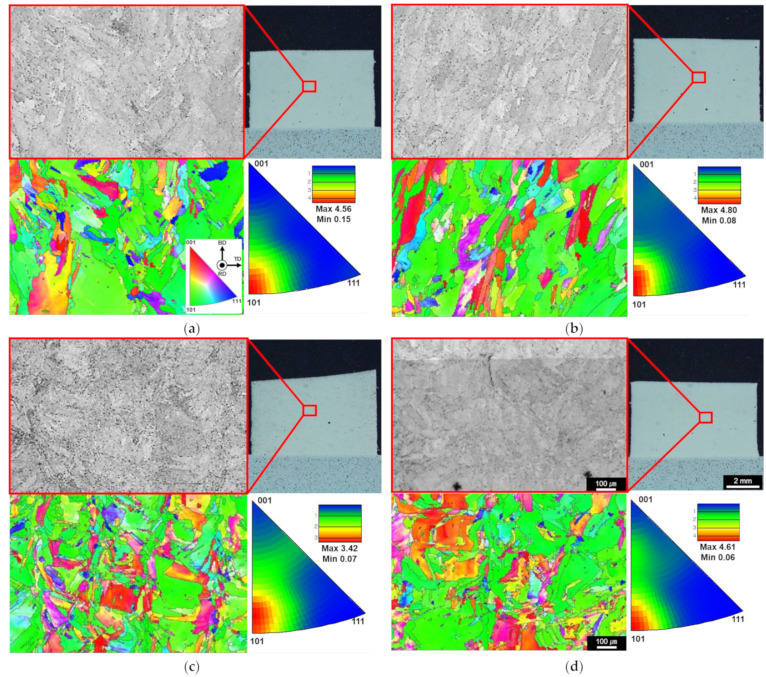
EBSD IPF maps and band contrasts along the BD direction for samples (**a**) Lo–U, (**b**) Lo–T, (**c**) LR–U, and (**d**) LR–T.

Figure 6 shows the EBSD inverse-pole figure (IPF) maps along the BD direction for the HMnS samples with the corresponding IPFs. The EBSD analysis showed that all samples had fully austenite, face-centered cubic phase microstructures. Regardless of the laser deposition strategies used, a strong <101>//BD texture is the main textural component observed for the HMnS samples. This indicates that the textural components are not significantly influenced by laser rescanning or the laser tool path. The texture intensity was varied from sample to sample in a range between 3.42 and 4.8, but the difference was not significant. In the IPF maps, elongated grain structures due to fast solidification are seen in the Lo–U and Lo–T samples. In the case of the LR–U and LR–T samples where the laser was rescanned on each layer, more complex grain structures were found, presumably due to the rapid solidification in the LR process, which occurs in a slightly different direction to that in the normal L-DED process [20,21]. The heat dissipation in the melt pool in the normal L-DED process occurs through the previously deposited layer underneath and also laterally on just one side of the melt pool though the already deposited laser bed. In the case of laser rescanning, lateral heat dissipation can occur in two sides of the melt pool through the already deposited dense material. For this reason, the solidification rate can increase, and the solidification direction can change in the LR in comparison to that in the normal L-DED process. Due to the higher solidification rate, the grain morphologies of the LR-U and LR-T samples became more equiaxed in comparison to those for the Lo-U and Lo-T samples.

The grain sizes measured from the EBSD results were 63.6, 66.5, 44.1, and 49.1 μm for the Lo–U, Lo–T, LR–U, and LR–T samples, respectively. It indicates that the grain size of the samples produced with LR is approximately 30% smaller than that in the samples without LR due to the faster solidification in the LR process. The deposited HMnS comprises full γ–austenite, without any ε– or α^′^–martensites. This was due to the strong austenite stabilization effect of Mn. A similar observation was made in the L-DED-processed HMnS with a manganese content of 12 wt.% [22]. For the HMnS with a manganese content higher than 24 % used in this study, Hufenbach et al. [25] reported that the Fe-30Mn-1C-0.02S (wt.%) alloy deposited through a selective laser melting process also comprises γ–austenite as the main phase.

### 3.2. Mechanical Properties

**Figure 7:**
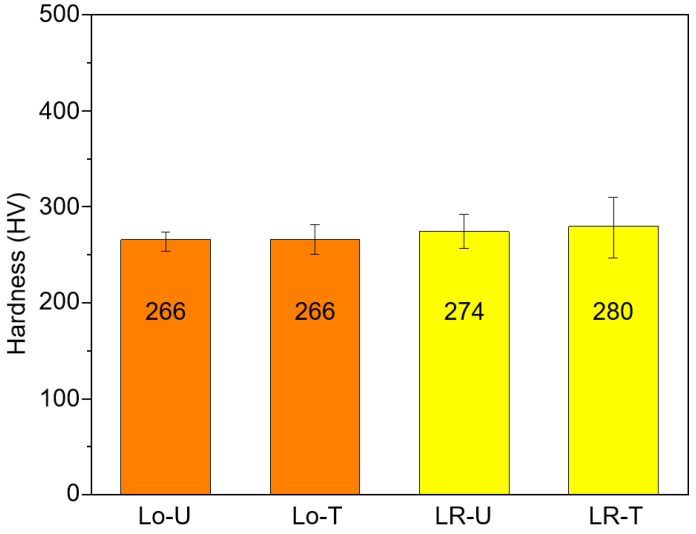
Vickers microhardness of HMnS samples produced with and without LR.

Figure 7 shows the microhardness results of the deposited HMnS with the Lo–U, Lo-T, LR-U, and LR–T scanning strategies. The sample produced only by the L-DED without LR showed ~266 HV. In the case of the sample produced with LR, the hardness was approximately 274~280 HV, which is ~4% higher than that in the sample without LR. 

**Figure 8:**
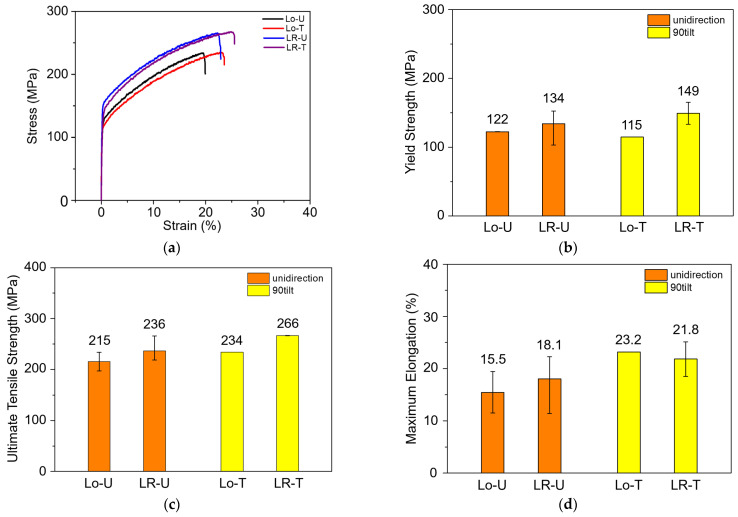
Tensile test results of HMnS samples: (**a**) Representative tensile stress–strain curves, (**b**) yield strength, (**c**) ultimate tensile strength, and (**d**) maximum elongation.

Figure 8 shows the tensile test results of the HMnS deposited by the L-DED process. Representative tensile stress–strain curves are shown in Figure 8a. The stress–strain curves of the HMnS produced by all four different laser strategies are characterized as typical ductile deformations. Generally, the error bars for the yield strength of the laser-rescanned samples showed larger deviations in comparison to that in the samples produced by L-DED only. Nevertheless, the results shown in Figure 8b,c clearly show that the samples with LR showed higher yield and tensile strengths than those for the samples produced by L-DED only. For both the cases, with and without LR, the samples produced with unidirectional laser scanning without a 90° alteration exhibit almost identical stress–strain curves, with only slightly higher yield strengths than for those produced with a 90° alteration in the laser tool path on each layer; that is, Lo–U exhibited a near-identical curve to that of Lo–T. The same trend was observed between the LR–U and LR–T samples. This indicates that the anisotropy of the mechanical properties between the laser scanning and its perpendicular direction of each L-DED layer is negligible for the HMnS. The bars and error bars in Figure 8b–d indicate averages and standard deviations of the yield strength, ultimate tensile strength, and maximum elongation, respectively. 

In comparison to the conventional hot-rolled and annealed HMnS, the L-DED-processed HMnS in this study showed relatively low strength and moderate elongation [26]. This is probably due to the relatively large grain size obtained from the L-DED process. It is known that the grain size-dependent yield strength of the HMnS clearly obeys the Hall–Petch relation, given as follows:

(1) $$\sigma_{ys}=\sigma_{ys}^{0}+\frac{k}{\sqrt{d}},$$

where $\sigma_{ys}$ is the yield strength in MPa, d is the average grain diameter in μm, and $\sigma_{ys}^{0}$ and k are constants. For the conventionally processed HMnS containing 17–25 wt.% Mn, the reported values of $\sigma_{ys}^{0}$ and k are 184 MPa and 433 MPa μm^−1^, respectively, in the case of fine-grained alloys with grain sizes less than 2 μm [26]. Substituting the experimentally obtained grain sizes of 49.1–66.5 μm of the HMnS produced by L-DED in Equation (1) gives the yield strengths in a range of 237–249 MPa. This is a significant overestimation, considering that the observed yield strengths of the L-DED-processed HMnS were approximately 115–149 MPa. One possible reason for this overestimation is the significantly larger grain sizes of the L-DED-processed HMnS than those of the hot-rolled and annealed alloys. The grain sizes of 49.1–66.5 μm of the HMnS produced by L-DED observed in this study are comparable to or even slightly larger than the grain size of ~50 μm of the HMnS prepared by casting [27]. The values of $\sigma_{ys}^{0}$ and k for the conventionally processed alloys in the wide range of grain sizes of 2.1–72.6 μm were reported by Dini et al. [28] as 53 MPa and 764 MPa μm^−1^, respectively. The predicted yield strengths from these values with Equation (1) for the Lo–U, Lo–T, LR–U, and LR–T are 149, 147, 168, and 162 MPa, respectively. It still slightly overestimates the yield strength of the HMnS produced by the L-DED, but the difference was not as significant as that with the predictions from the values for the fine-grained alloys. These predictions represent the experimentally observed strengthening effect, with the yield strength increased by approximately 20 MPa due to the grain refinement during LR.

**Figure 9:**
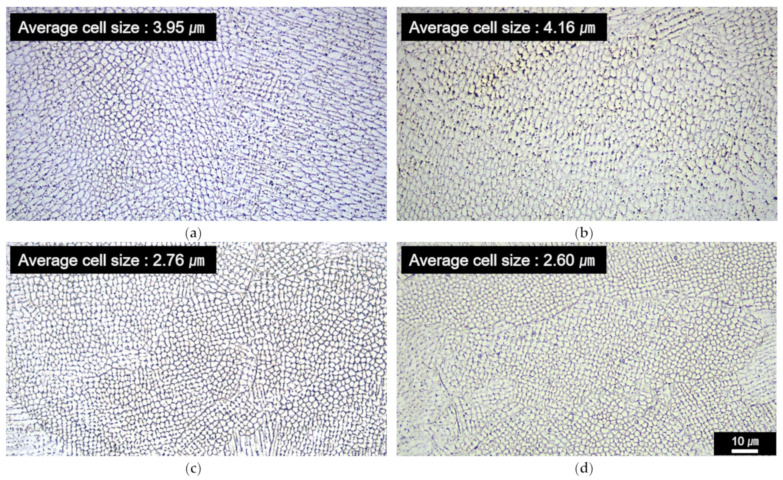
OM images of HMnS samples deposited with different laser strategies (×1000): (**a**) Lo–U, (**b**) Lo–T, (**c**) LR–U, and (**d**) LR–T.

The results shown in Figure 7 and Figure 8 indicate that both the microhardness and tensile strength increased with the LR. The analysis with the Hall–Petch relation showed that the grain refinement effect due to the LR is one reason for the increase in the microhardness and strength. Another possible reason could be the fine solidification cell size in the LR-processed HMnS in comparison to those processed by L-DED only without LR. Figure 9 shows OM images of the HMnS samples deposited with different laser strategies. The OM images were obtained carefully to capture the microstructures where the solidification cells were aligned near perpendicularly to the sample cross-section to observe the cell wall structures. Analysis of the OM images shown in Figure 9 by the ImageJ software indicated average cell sizes of 3.95, 4.16, 2.76, and 2.60 μm for samples Lo–U, Lo–T, LR–U, and LR–T, respectively. Similar results were found previously in the L-DED process of AISI 316L stainless steel due to the significantly faster solidification rate in LR than that in L-DED [21]. The finer solidification cell structures can also result in higher hardness and strength, which can also explain the improved mechanical properties through LR.

## 4. Summary

This study investigated the effect of laser rescanning on the microstructure and mechanical properties of laser-directed energy-deposited high-manganese steels. The samples were deposited by applying four types of laser deposition and LR strategies. The major findings are summarized as follows:

1. L-DED-processed high-manganese steel containing 24 wt.% Mn had a microstructure comprising solidification cell structures with a full face-centered cubic austenite phase. Near defect-free microstructures with low porosities ranging from 0.39 to 0.68% were produced. A strong <101>//BD texture was developed in the microstructures with and without laser rescanning.
2. The melt pool formed by laser rescanning was smaller than that produced in the L-DED process. The averaged melt pool heights of the samples with and without laser rescanning were approximately 184 and 75 μm, respectively. This was probably due to the relatively fast heat dissipation to the surrounding material in laser rescanning in comparison to that in L-DED. Both the grain and the solidified cell sizes were refined when the laser was rescanned after each layer of L-DED deposition.
3. Tensile test results indicated that the high-manganese steel produced by L-DED exhibits typical ductile deformations. In comparison to the conventional hot-rolled and annealed high-manganese steels, the L-DED-processed alloy exhibited a relatively low yield strength of approximately 115–149 MPa, with moderate elongation of 15.5–23.2%. This was believed to be from the relatively large grain size of the alloy obtained from the L-DED process. With laser rescanning, both the microhardness and tensile yield strength increased significantly.
4. Analysis through the Hall–Petch relation predicts a yield strength increasement of ~20 MPa due to the grain refinement resulting from laser rescanning. It was found that the solidified cell structure of the laser-rescanned samples was significantly finer than those produced without laser rescanning. Thus, the laser-rescanned high-manganese steels showed improved hardness and strength compared to those without laser rescanning due to the fine grain size and refined solidified cell size.

## Figures and Tables

**Table 1:** Process parameters used for L-DED and laser rescanning.

Process	Parameter	Value
L-DED	Laser power (W)	190
	Scanning speed (mm/min)	1080
	Powder feed rate (g/min)	1.5
	Hatch space (mm)	0.3
	Layer thickness (mm)	0.15
Laser Rescanning	Laser power (W)	190
	Scanning speed (mm/min)	1080

## Data Availability

The data that support the findings of this study are available from the corresponding author upon reasonable request.
